# Do Laparoscopic Approaches Ensure Oncological Safety and Prognosis for Serosa-Exposed Colon Cancer? A Comparative Study against the Open Approach

**DOI:** 10.3390/cancers15215211

**Published:** 2023-10-30

**Authors:** Ji-Hyun Seo, In-Ja Park

**Affiliations:** 1 Department of Surgery, Inha University Hospital, College of Medicine, Incheon 22332, Republic of Korea; jhseo@inha.ac.kr; 2 Department of Colon and Rectal Surgery, Asan Medical Center, University of Ulsan College of Medicine, Seoul 05505, Republic of Korea

**Keywords:** serosa-exposed, colon neoplasm, laparoscopy, seeding

## Abstract

**Simple Summary:**

This study examined whether laparoscopic surgery increases the risk of cancer recurrence in patients with T4a colon cancer. A total of 315 patients were analyzed, with 148 in the laparoscopic group and 167 in the open surgery group. Recurrence rates, patterns, and times were similar between the groups. While there was a slightly higher rate of peritoneal metastasis in the laparoscopic group, it was not statistically significant. The five-year overall survival (OS) and disease-free survival (DFS) were not significantly different between the two groups. Age and the pathologic N stage were associated with OS, and only the pathologic N stage was a risk factor for DFS. In conclusion, laparoscopic surgery does not compromise oncologic outcomes in T4a colon cancer patients, despite peritoneal seeding being a common type of recurrence.

**Abstract:**

The adoption of laparoscopic surgery in the management of serosa-exposed colorectal cancer has raised concerns. This study aimed to investigate whether laparoscopic surgery is associated with an increased risk of postoperative recurrence in patients undergoing resection for serosa-exposed colon cancer. A retrospective analysis was conducted on a cohort of 315 patients who underwent curative resection for pathologically confirmed T4a colon cancer without distant metastases at the Asan Medical Center between 2006 and 2015. Patients were categorized according to the surgical approach method: laparoscopic surgery (MIS group) versus open surgery (Open group). Multivariate analysis was employed to identify risk factors associated with overall survival (OS) and disease-free survival (DFS). The MIS group included 148 patients and the Open group had 167 patients. Of the total cohort, 106 patients (33.7%) experienced recurrence during the follow-up period. Rates, patterns, and time to recurrence were not different between groups. The MIS group (55.8%) showed more peritoneal metastasis compared to the Open group (44.4%) among recurrence sites, but it was not significant (*p* = 0.85). There was no significant difference in the five-year OS (73.5% vs. 78.4% *p* = 0.374) or DFS (62.0% vs. 64.6%; *p* = 0.61) between the Open and MIS groups. Age and the pathologic N stage were independently associated with OS, and the pathologic N stage was the only associated risk factor for DFS. The laparoscopic approach for serosa-exposed colon cancer did not compromise the DFS and OS. This study provides evidence that laparoscopic surgery does not compromise oncologic outcomes of patients with T4a colon cancer although peritoneal seeding is the most common type of disease failure of serosa-exposed colon cancer.

## 1. Introduction

There has been steady progress in the field of minimally invasive surgical treatments for colorectal cancer over the past decade [1,2,3,4,5,6,7,8]. Laparoscopic surgery has revolutionized the field of colorectal cancer treatment, gaining widespread acceptance and becoming a routine choice for many patients due to its feasibility and proven oncologic safety in numerous studies [1,2,3,5,7]. However, despite the generalized use of the laparoscopic approach, concerns continue to linger regarding the oncologic safety of laparoscopic surgery when applied to a specific subgroup of colon cancer patients: those with serosa-exposed tumors classified as pathologic T4a [9,10,11].

Notably, pivotal clinical trials and many previously reported clinical studies that have investigated the oncologic safety of laparoscopic surgery for colon cancer have consistently excluded patients with pathologic T4 tumors from their analyses, leaving a critical gap in our understanding of the suitability of this minimally invasive approach for this particular subset of patients [2,5]. Higher T stage is a risk factor for developing peritoneal disease due to direct seeding of malignant cells [12,13]. The apprehension surrounding laparoscopic surgery in serosa-exposed colon cancer arises from the potential dissemination of cancer cells, although mechanism is not clearly established, which could theoretically compromise long-term oncologic outcomes [8,9,10,14].

In recent years, as the adoption of laparoscopic surgery has continued to expand, several studies have emerged comparing the oncologic outcomes between laparoscopic and open surgery specifically for serosa-exposed (clinical or pathological T4a) colon cancer [15,16,17,18,19]. Some recent studies reported that laparoscopic surgery is associated with an increased risk of peritoneal recurrence compared with open surgery in T4 colon cancer [20,21]. However, some of these studies have presented a significant challenge in terms of heterogeneity within patient groups. Some studies have included patients in the clinical T4 stage, while others failed to clearly distinguish between T4a and T4b tumors [17,18,19]. Consequently, the oncologic safety of the minimally invasive approach for pT4a colon cancers remains uncertain.

In this context, we need to review the literature and analyze existing data thoroughly regarding the oncologic safety of laparoscopic surgery for serosa-exposed (clinical or pathological T4a) colon cancer. By addressing the current uncertainties surrounding this approach, we hope to guide clinical decision-making and enhance our understanding of the optimal surgical management approach for patients with pathologic T4a colon cancer.

This study aimed to analyze the oncologic outcomes of minimally invasive surgery (MIS) concerning the recurrence rate and type of recurrence of recurrence in resected pT4a colon cancer, comparing these findings with those obtained from open colectomy (Open).

## 2. Materials and Methods

### 2.1. Patient Population

**Figure 1:**
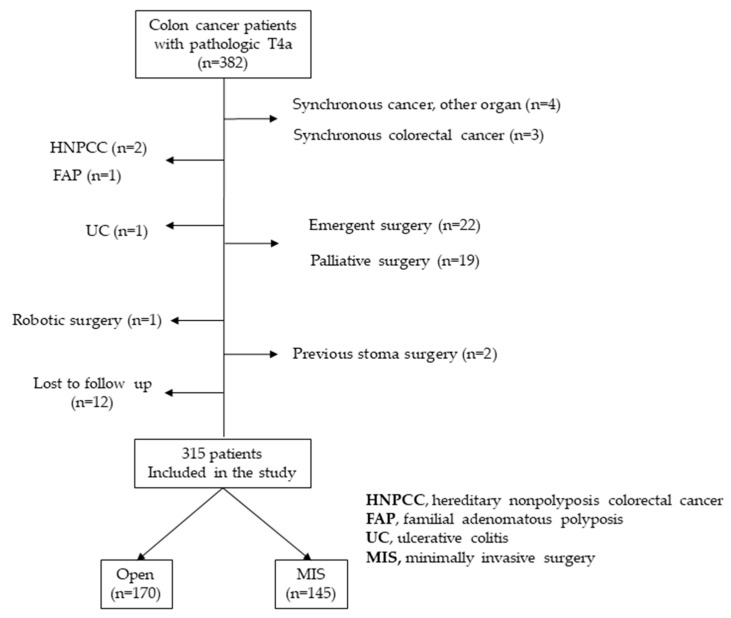
Inclusion and exclusion criteria for the overall cohort.

A total of 382 colon cancer patients with pathologic T4a colon adenocarcinoma underwent surgical treatment between January 2006 and December 2015, and they were included in the evaluation. Out of these patients, 67 patients were excluded; four had synchronous cancer of other organs, three had synchronous colorectal cancer, two had hereditary nonpolyposis colorectal cancer, one had familial adenomatous polyposis, one had ulcerative colitis, twenty-two required emergency surgery, one underwent robotic surgery, nineteen underwent palliative surgery, two had previous stoma surgery, and twelve were lost during follow-up immediately after surgery. As a result, 315 patients were included in the study (Figure 1).

The tumor location was classified into two categories: right colon (from the cecum to the transverse colon) and left colon (from the splenic flexure to the sigmoid colon). Any complications occurring within 30 postoperative days were evaluated. Resected tumors were staged according to corresponding TNM classification of colon cancer, proposed by the American Joint Committee on Cancer applicable for that specific period [13].

This was a single center (Asan Medical Center, Seoul, Republic of Korea) observational retrospective cohort analysis, and the study protocol was approved by the Institutional Review Board of the Asan Medical Center (registration number: 2020-0232), in accordance with the Declaration of Helsinki. The requirement for informed consent was waived due to the retrospective nature of this study.

### 2.2. Treatment, and Surveillance

All surgical procedures were performed by seven specialized colorectal surgeons. Radical resection with the principal of en-bloc removal of the primary tumor with lymph node dissection was performed. The selection of the type of surgical approach was usually decided by discussion between the surgeons and patients, considering the general condition, disease extent, and surgeon`s experience. Postoperative adjuvant chemotherapy was recommended for patients who were physically capable of receiving the treatment.

A comprehensive follow-up protocol was implemented for all patients in accordance with the institutional policy. The general practice for the postoperative surveillance program in our institute is as follows. Patients are followed normally at six-month intervals for the first two years after surgery and at six- or twelve-month intervals thereafter. At each visit, serum CEA levels are measured. Chest radiographs and abdominopelvic computed tomography (APCT) are performed six months after curative resection, and then at half-year intervals with chest radiographs and APCT and yearly intervals with chest CT. Colonoscopy was typically performed within six months to one year after surgery and then repeated every two years unless advanced adenomas were identified. Unplanned CT or positron emission tomography (PET) scans were performed on patients with increased serum CEA levels or patients who were symptomatic. Patients with suspicious but indeterminate findings on surveillance CT scans, magnetic resonance imaging (MRI), or PET scans were further evaluated based on recommendations from radiologists. The diagnosis of tumor recurrence was based on radiographic evidence of new lesions with clinical progression and/or histological confirmation through biopsy or examination of the resected specimen. The time to recurrence was confirmed by the date of the appearance of new lesions in imaging studies.

The type of recurrence was classified as systemic, loco-regional, and combined recurrence. Overall survival was defined as the duration from surgery to death from any cause. Disease-free survival was defined as the duration from surgery to disease recurrence.

### 2.3. Statistical Analysis

The clinicopathologic variables between groups were compared using the chi-square test or Fisher’s exact test for categorical variables and the student’s *t*-test for continuous variables. Overall survival (OS) and disease-free survival (DFS) were calculated from the date of surgery until the date of recurrence, death, or the last follow-up, compared using the Kaplan–Meier method with a log-rank test. Logistic regression analysis was used to evaluate the association between the clinicopathological risk factors and recurrence. Additionally, the Cox proportional hazards model was used to assess the prognostic effect of the risk factors on OS and DFS. Variables with *p*-value < 0.05 in univariate analysis were included in multivariate analysis. The confidence intervals (CI) were set at 95% and the statistical significance was defined as a *p*-value < 0.05. All statistical analyses were performed using SPSS software (version 21.0; IBM Statistics, Armonk, NY, USA).

## 3. Results

### 3.1. Clinicopathological Characteristics of Patients with pT4a Colon Cancer and Surgical Outcomes

Among the 315 included patients, the Open and MIS groups included 170 and 145 patients, respectively. Table 1 displays the clinicopathologic characteristics of the Open and MIS groups, revealing no significant difference in any clinicopathologic variables between the two groups. In the MIS group, six patients (4.1%) required conversion to open surgery. Of these cases, three conversions were attributed to severe adhesions, two were due to suspected invasion of other organs, and one was a result of iatrogenic damage to other organs. The proportion of patients who received adjuvant chemotherapy was 89.2% in the Open group and 87.8% in the MIS group (*p* = 0.62). FOLFOX was the most common regimen in entire cohort (n = 143, 51.3%) followed by XELODA (n = 59, 21.1%), XELOX (n = 51, 20.8%), and FL (n = 15, 5.4%). Other regimens including FOLFIRI, UFT, and S1 were used for eleven patients. Oxaliplatin-based chemotherapy (FOLFOX and XELOX) was used more in the MIS group (77.7%) than in the Open group (62.5%).

Postoperative morbidity was observed in 20 cases (6.3%), but no mortality occurred. The rate of postoperative complication was significantly higher in the Open group (fifteen cases, 9.0%) compared to the MIS group (five cases, 3.4%) (*p* = 0.04). In the Open group, there were four cases of ileus, two cases of anastomosis leakage, one case of bleeding, three cases of wound complications, three cases of intra-abdominal abscess formation, and two cases of retrograde ejaculation. In the MIS group, there were two cases of ileus, one case of cerebral infarction, and two cases of intra-abdominal abscess formation. Patients with higher than Clavien–Dindo classification III were also more common in Open group (4.8%) than in the MIS group (n = 0.7%) (*p* = 0.039).

### 3.2. Recurrence Pattern According to Type of Approach

Among the 315 patients, a total of 106 (33.7%) experienced recurrence. Recurrence included 97 cases of systemic recurrence, seven cases of loco-regional recurrence, and two cases of combined recurrence. The time to recurrence did not differ significantly between the two groups (Open: 17.7 ± 9.1 months, MIS: 19.2 ± 10.1 months, *p* = 0.64).

The rates and pattern of recurrence were compared between the Open and MIS groups. In the Open group, 54 patients experienced systemic recurrence. The most common metastasis was peritoneal seeding (n = 24, 44.4%), followed by liver (n = 12, 22.2%), lung (n = 9, 16.7%), ovary (n = 4, 7.4%), distant lymph node (n = 4, 7.4%), and brain (n = 1, 1.9%) metastasis. In the MIS group, 43 patients had systemic recurrence. Peritoneal seeding was also the most common type of metastasis (n = 24, 55.8%). This was followed by liver (n = 8), lung (n = 7), distant lymph node (n = 2), and ovary metastasis (n = 2). No statistically significant difference was observed in the distribution of recurrent organs between the two groups (*p* = 0.85). Notably, peritoneal seeding was the most frequently encountered metastasis in both the Open and MIS groups, accounting for 49.5% of systemic recurrence in both groups.

In female patients, ovary metastasis occurred at a rate comparable to that of liver and lung metastasis. Among the 72 women in the Open group, 31 experienced systemic recurrence, with ovary metastasis observed in four patients (12.9%). Among 74 women in the MIS group, 24 had systemic recurrence, and ovary metastasis occurred in two patients (8.3%). This was comparable to the incidence of lung and liver metastasis (Table 2).

### 3.3. Overall Survival, Disease-Free Survival, and Associated Factors

**Figure 2:**
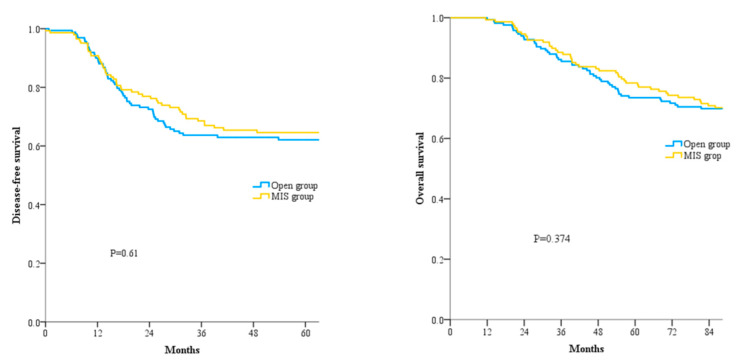
Disease-free survival (**left**) and overall survival (**right**) according to surgical approach. The MIS group did not differ from the Open group in either disease-free survival or overall survival.

The mean follow-up duration was 97.5 ± 44.3 months. In the cohort of patients with pT4a colon cancer, the five-year OS rate was 75.8% and the five-year DFS rate was 63.2%. There was no significant difference in the five-year OS rate (73.5% in the Open group vs. 78.4% in the MIS group) or DFS rate (62.0% in the Open group vs. 64.6% in the MIS group) between the two groups (Figure 2).

For DFS, the advanced pathologic N stage was only an associated factor (Table 3). Old age and the pathologic advanced pathologic N stage were independently associated with OS (Table 4).

Considering the high frequency of peritoneal seeding, we conducted an analysis of peritoneal seeding-free survival and identified risk factors associated with peritoneal seeding. The five-year peritoneal seeding-free survival of the entire cohort of T4a colon cancer was 80.8%. There was no significant difference in the five-year peritoneal seeding-free survival between the Open and MIS groups (OC 81.4% vs. 80.3%, *p*-value = 0.77). The Cox proportional multivariate hazard model showed that the surgical approach was not associated with peritoneal seeding-free survival (Table 5). The surgical method used was not identified as a risk factor for OS, DFS, or peritoneal seeding-free survival.

## 4. Discussion

In this study, our findings indicated that the oncologic outcomes, including the rate of overall and disease-free survival, recurrence rates, types of recurrence, and time to recurrence, were comparable between patients with pT4a colon cancer who underwent minimally invasive surgery (MIS group) and those who underwent open colectomy (Open group). Laparoscopic surgery was not associated with peritoneal metastasis according to the results of this study.

The surgical approach was chosen based on the clinical stage, but it was not usually correlated well with the pathologic stage. Overstaging due to peritumoral inflammation, particularly in cases of microsatellite instability, is a challenging problem. In addition, to mitigate the risk of underestimation, minimal pericolic fat stranding, caused by a benign desmoplastic reaction, might be interpreted as if it were tumor invasion [22,23]. The use of laparoscopic approaches has expanded to more advanced stages, and there has been a rise in the number of patients diagnosed with pT4a postoperatively. However, if there is a concern that laparoscopic surgery for T4 cancer could elevate the risk of tumor seeding in the port-site or peritoneum [24], we consider laparoscopic procedures for these patients carefully. In addition, the recurrence pattern might also influence the postoperative surveillance method.

Peritoneal recurrence after curative resection of advanced colon cancer has been reported in 2–10% of patients [25,26]. In contrast to the typical recurrence pattern observed in colon cancer patients [26,27], peritoneal seeding is found to be the most common form of recurrence in pT4a colon cancer cases [12,13,15]. The impact of a laparoscopic approach on peritoneal recurrence in cases of serosa-exposed colon cancer has been reported inconsistently in previous studies [15,16,17,19,28]. In our current study, we found that the recurrence pattern and time to recurrence were similar regardless of the surgical approach. While peritoneal seeding occurred more frequently in cases of recurrence, the proportion of peritoneal seeding was comparable between the laparoscopic and open surgery groups. The exact mechanism behind the development of peritoneal disease after laparoscopic surgery remains unclear, but several hypotheses have been considered. These include tumor seeding as a result of changes induced by pneumoperitoneum [28] or manipulation of the tumor using laparoscopic instruments, which could lead to tumor breakage and seeding. However, it’s worth noting that the importance of minimizing non-direct manipulation of the tumor during laparoscopic surgery is well recognized and emphasized in current practice. This approach helps mitigate the risk of peritoneal tumor spread associated with handling. While further research is needed to determine the precise influence of laparoscopic surgery on peritoneal recurrence, we advise caution when selecting the operative approach for patients with a perceived higher risk of developing intraperitoneal tumor spread.

Interestingly, ovarian metastasis is found to be highly prevalent among women with pT4a colon cancer, with the incidence of liver and lung metastasis being similar in this study. In the diagnosis of ovarian lesions, ultrasound is generally considered the most useful diagnostic method [29]. Consequently, the current practice of utilizing abdominopelvic CT as the primary follow-up method in women may not be optimal. It is worth considering the inclusion of trans-vaginal ultrasound as an additional follow-up test for the early detection of ovarian metastasis in female patients with T4a colon cancer.

In our study, LN metastasis was found to be a significant risk factor for OS and DFS. Consistent with our findings, some researchers have reported the safety of laparoscopic surgery for T4a colon cancer [8,16,30]. The laparoscopic approach was not related with poor DFS, OS, or PSFS. Previous studies have reported that the utilization of the non-touch technique during surgery leads to a reduced rate of tumor cell detection in the draining vein and portal venous system [31,32]. As a result, laparoscopic surgery offers advantages in terms of tumor control and long-term survival, contrary to theoretical risk of the laparoscopic approach.

The higher incidence of postoperative complications observed in patients who underwent open colectomy compared to those who underwent MIS could potentially be attributed to the advantages offered by MIS. These advantages include reducing pain, improving pulmonary function, reducing recovery time for bowel function, improving quality of life, and improving convalescence [1,2,5,8,33,34]. However, it is important to consider the possibility of selection bias in our study, which may have influenced the distribution of patients in the Open and MIS groups. We assumed that the patient groups were comparable in terms of clinical characteristics, given the similarities in age, BMI, and ASA score distributions between the two groups. However, we acknowledge that we were unable to comprehensively collect data on medical co-morbidities and directly compare the patients’ medical conditions, which could potentially influence post-surgical complications. In addition, it is possible that more severe and aggressive cancers were included in the Open group, which could contribute to the higher incidence of postoperative complications. This bias should be taken into account when interpreting the results and considering the potential benefits of MIS in reducing complications.

This study has some limitations. It has a retrospective design and included patients from a single tertiary center, introducing the potential for referral and selection biases. However, due to the challenges in accurately diagnosing pT4a colon cancer before surgery, randomization prior to surgery could be challenging. Given these difficulties, conducting retrospective research using large cohorts is currently a practical approach. In addition, the surgical and oncological results presented in our study are derived from a tertiary hospital with highly specialized surgeons. Therefore, there may be limitations in the generalizability of the findings to other healthcare settings. Acknowledging these limitations, our study contributes valuable insights into the outcomes of pT4a colon cancer patients. Further research addressing these limitations and utilizing multicenter prospective designs would provide additional robust evidence in this field.

## 5. Conclusions

In conclusion, our study demonstrated that the MIS approach for serosa-exposed colon cancer did not lead to an increased risk of tumor recurrence, indicating that it does not compromise the long-term prognosis of patients. Although the MIS group showed a higher rate of peritoneal metastasis compared to the Open group, this difference was not statistically significant. Further investigation is required to better understand the impact of MIS on peritoneal metastasis. In addition, surveillance protocols based on recurrence pattern in serosa-exposed colon cancer have to be evaluated for optimal detection of recurrence.

## Figures and Tables

**Table 1:** The clinicopathological characteristics of patients with pT4 colon cancer.

Characteristic	Open Group (n = 167)	MIS Group (n = 148)	*p*
Age (years)			0.61
≤65	104 (62.3%)	88 (59.5%)	
>65	63 (37.7%)	60 (40.5%)	
Sex			0.22
Male	95 (56.9%)	74 (50.0%)	
Female	72 (43.1%)	74 (50.0%)	
BMI (kg/m^2^)			0.88
≤25	116 (69.5%)	104 (70.3%)	
>25	51 (30.5%)	44 (29.7%)	
ASA score			1
1–2	162 (97.0%)	144 (97.3%)	
3–5	5 (3.0%)	4 (2.7%)	
Tumor location			0.22
Proximal	94 (56.3%)	73 (49.3%)	
Distal	73 (43.7%)	75 (50.7%)	
Differentiation			0.40
WD/MD	135 (80.8%)	125 (84.5%)	
PD/Mucinous/SRC	32 (19.2%)	23 (15.5%)	
Clinical T stage			0.25
T1–2	10 (6.0%)	14 (9.5%)	
T3–4	157 (94.0%)	134 (90.5%)	
Clinical N stage			0.68
N−	37 (22.2%)	30 (20.3%)	
N+	130 (77.8%)	118 (79.7%)	
Pathologic N stage			0.90
N0	66 (39.5%)	56 (37.8%)	
N1	69 (41.3%)	65 (43.9%)	
N2	32 (19.2%)	27 (18.2%)	
Lymphovascular invasion	79 (47.3%)	97 (65.5%)	0.001
Perineural invasion	63 (37.7%)	71 (48.0%)	0.069
Adjuvant chemotherapy			0.45
Received	148 (88.6%)	127 (85.8%)	
Not received	19 (11.4%)	21 (14.2%)	

MIS, minimally invasive surgery; BMI, body mass index; ASA, American Society of Anesthesiology; WD, well differentiatied; MD, moderately differentiated; PD, poorly differentiated; SRC, signet ring cell.

**Table 2:** Surgical approach and recurrence in women.

	Open (n = 72)	MIS (n = 74)
Overall recurrence	33	24
Type of recurrence		
Systemic	31	24
Brain	1 (3.2%)	0
Lung	3 (9.7%)	4 (16.7%)
Liver	7 (22.6%)	5 (20.8%)
Systemic LN	1 (3.2%)	1 (4.2%)
Peritoneal seeding	15 (48.4%)	12 (50.0%)
Ovary	4 (12.9%)	2 (8.3%)
Locoregional	1	0
Combined	1	0

CI, confidence interval; HR, hazard ratio.

**Table 3:** Factors associated with disease-free survival in pT4a colon cancer.

	Univariate Analysis	
Variables	Hazard Ratio (95%CI)	*p*
Age (yrs)		0.92
≤65		
>65	0.98 (0.66 -1.45)	
Sex		0.44
Male		
Female	1.48 (1.01–2.17)	
BMI (kg/m^2^)		0.55
≤25		
>25	0.88 (0.58–1.34)	
ASA score		0.8
1–2		
3–5	0.86 (0.27–2.71)	
Tumor location		0.64
Proximal		
Distal	1.09 (0.75–1.60)	
Differentiation		0.39
WD/MD		
PD/Mucimous/SRC	0.79 (0.47–1.35)	
Clinical T stage		0.45
T1–2		
T3–4	1.35 (0.63–2.90)	
Clinical N stage		0.8
N−		
N+	0.94 (0.60–1.49)	
Pathologic N stage		0.02
N0		
N1	1.78 (1.12–2.83)	
N2	2.52 (1.48–4.29)	
Lymphovascular invasion		0.26
No		
Yes	1.17 (0.89–1.55)	
Perineuarl invasion		0.95
No		
Yes	0.98(0.71–1.26)	
Surgical approach		0.61
Open		
MIS	0.90 (0.62–1.33)	
Adjuvant chemotherapy		0.21
Not received		
Received	0.71 (0.42–1.22)	

CI, confidence interval; HR, hazard ratio; BMI, body mass index; ASA, American Society of Anesthesiology; WD, well differentiated; MD, moderately differentiated; PD, poorly differentiated; SRC, signet ring cell; MIS, minimally invasive surgery.

**Table 4:** Factors associated with overall survival in pT4a colon cancer.

	Univariate Analysis		Multivariate Analysis	
Variables	Hazard Ratio (95%CI)	*p*	Hazard Ratio (95%CI)	*p*
Age (yrs)		0.004		0.005
≤65				
>65	1.74 (1.19–2.52)		1.75 (1.18–2.60)	
Sex		0.45		
Male				
Female	1.16 (0.79–1.68)			
BMI (kg/m^2^)		0.83		
≤25				
>25	0.96 (0.64–1.44)			
ASA score		0.04		0.12
1–2				
3–5	2.39 (1.05–5.46)		1.97(0.83–4.68)	
Tumor location		0.56		
Proximal				
Distal	0.89 (0.61–1.30)			
Differentiation		0.23		
WD/MD				
PD/Mucimous/SRC	0.72 (0.42–1.24)			
Clinical T stage		0.54		
T1–2				
T3–4	0.82 (0.43–1.57)			
Clinical N stage		0.14		
N−				
N+	0.72 (0.47–1.11)			
Pathologic N stage		0.02		0.04
N0				
N1	1.67 (1.07–2.59)		1.68 (1.04–2.70)	
N2	1.97 (1.18–3.31)		1.95 (1.13–3.37)	
Lymphovascular invasion		0.05		0.13
No				
Yes	1.47(1.00–2.16)		1.38(0.91–2.08)	
Perineural invasion		0.12		
No				
Yes	1.35(0.93–1.96)			
Surgical approach		0.38		
Open				
MIS	0.84 (0.58–1.23)			
Adjuvant chemotherapy		0.01		0.47
Not received				
Received	0.53 (0.33–0.85)		0.72 (0.38–1.33)	

CI, confidence interval; HR, hazard ratio; BMI, body mass index; ASA, American Society of Anesthesiology; WD, well differentiated; MD, moderately differentiated; PD, poorly differentiated; SRC, signet ring cell; MIS, minimally invasive surgery.

**Table 5:** Factors associated with peritoneal seeding-free survival.

	Univariate Analysis	
Variables	Hazard Ratio (95%CI)	*p*
Age (yrs)		0.87
≤65		
>65	0.95 (0.53–1.71)	
Sex		0.51
Male		
Female	1.21 (0.68–2.17)	
BMI (kg/m^2^)		0.73
≤25		
>25	1.12 (0.60–2.07)	
ASA score		0.41
1–2		
3–5	0.54 (0.12–2.36)	
Tumor location		0.09
Proximal		
Distal	0.61 (0.34–1.09)	
Differentiation		0.33
WD/MD		
PD/Mucimous/SRC	1.44 (0.69–2.99)	
Clinical T stage		0.25
T1–2		
T3–4	20.3 (0.62–6.70)	
Clinical N stage		0.71
N−		
N+	0.88 (0.44–1.74)	
Pathologic N stage		1
N0		
N1	1.00 (0.49–2.04)	
N2	1.00 (0.42–2.39)	
Lymphovascular invasion		0.68
No		
Yes	0.88(0.49–1.59)	
Perineural invasion		0.14
No		
Yes	0.63(0.35–1.16)	
Surgical approach		0.98
Open		
MIS	1.00 (0.56–1.80)	
Adjuvant chemotherapy		0.13
Not received		
Received	0.56 (0.27–1.18)	

CI, confidence interval; HR, hazard ratio; BMI, body mass index; ASA, American Society of Anesthesiology; WD, well differentiated; MD, moderately differentiated; PD, poorly differentiated; SRC, signet ring cell; MIS, minimally invasive surgery.

## Data Availability

The data presented in this study are available on request from the corresponding author. The data are not publicly available due to conditions of the ethics committee of our university.
